# A Mouse Model of Heritable Cerebrovascular Disease

**DOI:** 10.1371/journal.pone.0015327

**Published:** 2010-12-31

**Authors:** Thomas J. Sproule, John G. Sled, Jill Wentzell, Bing Wang, R. Mark Henkelman, Derry C. Roopenian, Robert W. Burgess

**Affiliations:** 1 The Jackson Laboratory, Bar Harbor, Maine, United States of America; 2 Hospital for Sick Children, University of Toronto, Toronto, Canada; RIKEN Brain Science Institute, Japan

## Abstract

The study of animal models of heritable cerebrovascular diseases can improve our understanding of disease mechanisms, identify candidate genes for related human disorders, and provide experimental models for preclinical trials. Here we describe a spontaneous mouse mutation that results in reproducible, adult-onset, progressive, focal ischemia in the brain. The pathology is not the result of hemorrhage, embolism, or an anatomical abnormality in the cerebral vasculature. The mutation maps as a single site recessive locus to mouse Chromosome 9 at 105 Mb, a region of shared synteny with human chromosome 3q22. The genetic interval, defined by recombination mapping, contains seven protein-coding genes and one processed transcript, none of which are changed in their expression level, splicing, or sequence in affected mice. Targeted resequencing of the entire interval did not reveal any provocative changes; thus, the causative molecular lesion has not been identified.

## Introduction

Hereditary vascular and cerebrovascular diseases such as Hereditary Hemorrhagic Telangiectasia (HHT), Cerebral Cavernous Malformation (CCM), and Cerebral Autosomal Dominant Arteriopathy with Subcortical Infarcts and Leukoencephalopathy (CADASIL) are clinically significant in their own right and also provide insights into the molecular mechanisms underlying blood vessel formation and integrity. Through the identification of such mutations, the roles of TGF-beta signaling (HHT) [1], [2], [3], [4], integrin pathways (CCM) [5], [6], [7], [8], [9], and Notch signaling (CADASIL)[10], [11] have been clearly demonstrated to be central to vascular biology. The brain is often the affected organ in such diseases. This sensitivity may reflect the complete dependence of the nervous system on aerobic respiration and also the complex relationship between the endothelial cells, pericytes, astrocytes and neurons comprising the neurovascular unit.

Animal models of such hereditary vascular diseases are needed for mechanistic studies and are also of interest in stroke research as a model of vascular impairment without the confound of surgical intervention. Therefore, the identification of new animal models of heritable vascular diseases is important for research, and for identifying candidate disease genes for related human conditions.

We have identified a spontaneous mutation in mice that leads to adult onset, bilateral, progressive cerebral ischemia. We have termed these mice “Decrepit” or *dcr* for their unhealthy appearance and shortened lifespan. The affected mice do not show evidence of clotting (embolism), hemorrhage (aneurism), or anatomical abnormalities in the cerebral vasculature. The mutation is inherited as a single recessive locus on mouse Chromosome 9, between 104.684 and 105.457 Mb, an interval containing seven genes and one processed transcript. These genes, and the region of shared synteny in the human genome, are therefore candidates for human hereditary vascular diseases.

## Methods

### Animal studies

All studies were performed in accordance with the Guide for the Care and Use of Laboratory Animals, and all procedures were approved by The Jackson Laboratory Animal Care and Use Committee, comprehensive protocol #01026, approval date Nov. 29, 2007. Strains, ages, and the number of animals examined in given experiments are provided in the text and figure legends at appropriate points. Animals were provide food (NIH 6% diet) and water ad libitum and were kept on a standard 14-10 light-dark cycle.

### Histology and immunocytochemistry

Standard histological staining techniques were used to analyze the pathology of the *dcr* mice. Tissue was fixed in Bouin's fixative, and was dehydrated and paraffin embedded using an automated tissue processor in the Histology and Light Microscopy Service at The Jackson Laboratory. Four µm paraffin sections were then cut using a microtome and collected onto slides for staining. Standard hematoxylin and eosin staining, phosphotungstic acid hematoxylin, and hemosiderin staining protocols were used. Sections stained for anti-glial fibrilary acidic protein (GFAP) were processed equivalently, but stained with a rabbit polyclonal antibody against GFAP (Sigma, St. Louis, MO, USA) and an HRP-conjugated anti-rabbit secondary antibody (Vector Labs, Burlingame, CA, USA). The signal was developed with diaminobenzidine (DAB) and nuclei were counter stained with hematoxylin (Sigma, St. Louis, MO, USA).

### Micro-CT imaging

Mice were anesthetized and perfused transcardially with yellow Microfil resin (Flow Tech, Inc., Carver, MA, USA), a radio-opaque silicone rubber containing particulate lead chromate and lead sulfate, and known for minimal shrinkage [12]. The resin was allowed to set and the entire mouse was fixed by immersion in 4% buffered formalin. The brain was subsequently dissected free of the skull and embedded in low melting point agarose. Micro-CT images were acquired in a 2 hour protocol in which 720 views were obtained through 360° rotation with a GE eXplore Locus SP Specimen Scanner using peak voltage 80 kVp, current 80 A [13]. The micro-CT images were reconstructed with isotropic cubic voxels of size 20 µm.

### Confocal imaging

Confocal reconstructions of the penetrating vessels and capillaries of the brain were obtained by breeding the *dcr* mutation to transgenic mice expressing GFP driven by the Tie2 (Tek) promoter (The Jackson Laboratory strain number JR3658), which is specifically expressed in blood vessels and myeloid hemopoietic cells [14]. These mice were then anesthetized and fixed by perfusion with 4% buffered paraformaldehyde. The brain was then dissected free and cut into 1 mm coronal sections using a matrix slicer and Teflon coated razor blade. Sections were immersed in PBS and imaged using a Leica SP2 confocal microscope with a 20X objective lens. Serial Z-sections were collected in 1 µm steps from the surface of the section to a depth of 50 µm. Projections of the Z-stacks are shown. Images were collected from the cortex near the infarcted regions. At least 4 stacks were collected for each mouse. Five *dcr* and four littermate control mice were examined. Mutant mice ranged from 72 to 103 days of age.

### Genetic mapping

The mutant decrepit (*dcr*) phenotype was first identified as wasting and early death in some but not all mice in the double congenic stock B6-H60^c^ Cd45^sjl^ produced by crossing B6.CBy-*H60a^c^*/2Dcr to B6.SJL-*Ptprc^a^ Pepc^b^*/BoyJ. *dcr* was then transferred onto and bred homozygously on a pure C57BL/6J genetic background. Mapping was carried out using an F2 cross of (BALB/cByJ x B6-*dcr/dcr*) and a backcross of ((NOD x B6-*dcr/dcr*) xB6-*dcr/dcr*)) mice. For finer mapping, B6-*dcr/dcr* mice were bred to B6 background BALB congenics originally derived from B10.C-*H7^b^*/(47N)Sn (strain JR430) which spanned the *decrepit* candidate interval, then tested for reduced BALB congenic segments that correlated with either retention of a healthy phenotype or conversion to the *decrepit* phenotype. All Mb positions are based on NCBI build 37, as accessed via the UCSC In-Silico PCR website (http://genome.ucsc.edu/cgi-bin/hgPcr?org=Mouse&db=mm8&hgsid=92079956). All genotyping was by simple sequence length polymorphism (SSLP). Initial localization to Chr. 9 was based on a genome-wide scan for linkage using *Mit* markers. For finer mapping, a series of *D9Dcr* primers (the most informative primers are listed below, additional markers in the interval can be found on the Mouse Genome Informatics website - www.informatics.jax.org/) were designed using Ensembl sequence such that primer pairs spanned regions of dinucleotide repeats. All primers were designed with 60 degree Centrigrade annealing temperatures, with products of ∼100 bp. About 40% of primer pairs produced in this way included usable B6 vs. BALB polymorphisms.

D9Dcr56F: TTTAAGACTTTATTCATGTGTCTCCAAAA

D9Dcr56R: GAAGCATCACATAAAACGTGTGTGT

(104.684 Mb, equivalent of *D9Mit53*)

D9Dcr70F: ACGAGTGGGCCAGTGAGTTCT

D9Dcr70R: CACTCATGGCTGTAAGCTTCGTA

(105.262 Mb)

D9Dcr75F: GCTGGCTATAAATACCTACATTAAGAGTGT

D9Dcr75R: TGCATAGTTAGAGATGACCTTGAACA

(105.382 Mb)

D9Dcr76F: AAAGACTGAGTGTGTGGAGTTGATCT

D9Dcr76R: TTACAGACTTCCCTTCCTTACATTAACA

(105.457 Mb)

### Candidate gene analysis

Genes were examined for their expression level, splicing and sequence. RNA from the brain of affected (approximately three month old) *dcr* mice was prepared using Trizol reagent (Invitrogen, Carlsbad, CA, USA). Total RNA (5 µg) was then used for reverse transcription using a mix of oligo-dT and random hexamer primers and Superscript III reverse transcriptase (Invitrogen, Carlsbad, CA, USA). This first-strand cDNA was used for Syber Green QPCR (Qiagen, Valencia, CA, USA) analysis on an ABI7900 (Applied Biosystems, Calsbad, CA, USA). Alternatively the cDNA was used as a template for amplification of coding sequences and sequencing of the PCR products. The RNA was also subject to poly-A+ selection (20 mg of oligo-dT per 500 µg of total RNA, Ambion, Austin, TX, USA) and northern blotting using PCR amplified probes for each gene in the *dcr* interval. Northern blotting was done by standard procedures using randomly primed alpha-dCT^32^P labeled probes. Signals were detected and quantified using both a phosphoimager (Fujifilm, Tokyo, Japan) and film (Kodak, Rochester, NY, USA). Blots were run in triplicate and at least three mutant and three control samples were on each blot.

### Array capture and targeted resequencing

Genomic DNA was prepared from the spleen of an affected *dcr* male mouse using phenol chloroform extraction and isopropanol precipitation. The DNA was then sent to Nimblegen for array capture (Nimblegen, Madison, WI, USA). A microarray with 385,000 features was created containing 60 bp tiled synthetic oligos spanning the 772 kb *dcr* genetic interval. The array was designed and printed by Nimblegen and repeat sequences were masked, leaving 654,907 bases of the interval on the array. The genomic DNA was then fragmented, hybridized to the array, and eluted. The eluted sample was prepared for Illumina sequencing by the Vanderbilt University Microarray Shared Resource. From the sequencing, a total of 7,434,305 reads were obtained (36 bp non-paired end), of which 3,419,255 mapped to the Chr. 9 interval using MAQ software (downloadable at http://sourceforge.net/projects/maq/files/), confirming excellent enrichment from the array capture. Only 1227 base pairs had read depths less than 3, and only 425 bases included on the array were not sequenced at least once. Computational analysis for single nucleotide polymorphisms (SNPs) using MAQ software detected four putative SNPs. All of these were shown to be errors in the reference genome when PCR amplified and resequenced by Sanger methods.

## Results

### Overt phenotype of *dcr* homozygous mice

**Figure 1:**
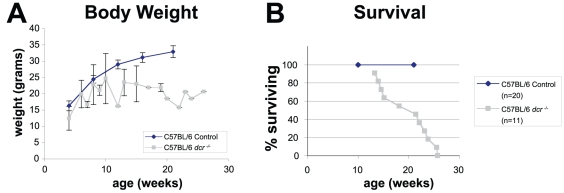
Growth and Mortality curves of *dcr* homozygous mice. A) The body weights of 20 control C57BL/6J male mice and 6 C57BL/6J *dcr*/*dcr* male mice are shown (mean ± SD). The body weights of the affected males begin to significantly diverge from controls at about 12 weeks of age. Gradual wasting is seen as the mice age. B) The survival of 11 C57BL/6J *dcr*/*dcr* mice is shown. Mice begin dying at 3 months of age. By 6 months, all eleven affected mice were dead. Control C57BL/6J mice from the same colony show no mortality in the same period. Note the number of mice in the body weight graph decreases with time due to mortality; however, at 12 weeks of age when the mutant curve diverges, all mice were alive.

Mice with the decrepit phenotype are normal at birth and for the first two months of life. At approximately 10 weeks of age, the mice begin to look unkempt, with a scruffy coat and abnormally hunched posture. Once affected, the mice are behaviorally abnormal as well, being initially excitable in response to stimulation and then lethargic (after approximately 15 weeks of age). The mice cease to gain weight and begin wasting after approximately 12 weeks of age (Figure 1A). Most mice succumb to the condition between 18 and 22 weeks of age, a survival curve for 11 males is shown in Figure 1B. Male and female mice are equally affected.

### Histopathology of *dcr* homozygous mice

**Figure 2:**
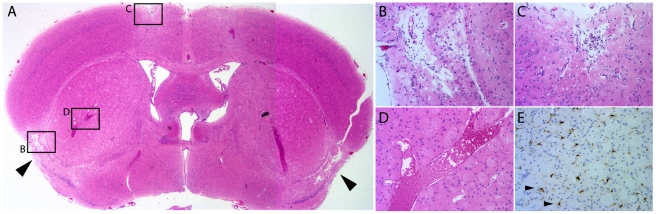
Pathology in the brain of *dcr* mice. A) A coronal section of a *dcr* mouse indicates bilaterally symmetric loss of neurons in the lateral, inferior cerebral cortex (arrowheads). B) The tissue loss is consistent with a regional ischemia resulting in complete vacuolization of the tissue in the affected region with normal surrounding tissue. C) Other regions of the brain such as the dorsal medial cortex eventually become affected. D) Dilated blood vessels are often present in the brains of affected mice. E) Activated astrocytes in the striatum stained for GFAP indicate stress reactions in regions of the brain that do not show obvious pathology (Arrowheads). Note that E is from additional sectioning of the same sample in a region comparable to D.

Full necropsies of five overtly affected, late-stage *dcr* mice were performed in consultation with a veterinary pathologist at The Jackson Laboratory. No pathology was seen in any organ system except the brain. In the brain, completely vacuolated regions were seen in the ventral cortex (Figure 2A). These regions were contiguous with the pial surface of the brain and were bilaterally symmetric. Consistent with an infarct, the vacuolated area was bounded by normal tissue (Figure 2A, B). Other brain regions were also affected, including the ventral hippocampus (not shown) and the perisagital dorsal cortex (Figure 2C). Severely dilated blood vessels were frequently observed, although such structures may also represent fixation artifacts (Figure 2D). Regions of the brain that did not show obvious pathology with standard histological stains did show signs of stress; for example, GFAP-positive reactive astrocytes were observed in the striatum (Figure 2E). This reaction in apparently normal brain regions may represent a response to degenerating axonal projections from other affected brain regions, or may be presaging eventual degeneration in other brain regions.

Despite pathology consistent with an infarct, we were unable to find evidence of typical stroke mechanisms such as hemorrhage or emboli. These were sought by serial sectioning through areas of recent damage, determined by the presence of dying neurons with eosin-rich cytoplasm and pyknotic nuclei, followed by phosphotungstic acid hemotoxylin (PTAH) and hemosiderin staining. In such areas, no PTAH-positive clots or hemosiderin-positive evidence of hemorrhage were found. Furthermore the bilateral symmetry of the damage makes such a mechanism less likely.

### Degeneration is progressive

**Figure 3:**
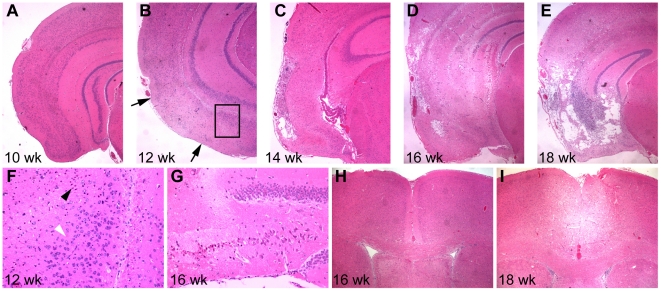
The pathology of the *dcr* locus is progressive. A time course was performed examining five mice at each of five time points from 10 to 18 weeks (only four study mice survived to 18 weeks). A) At ten weeks, none of the mice showed obvious pathology in the inferior lateral cortex. B) By twelve weeks, four out of five mice showed detectable pathology in the inferior lateral cortex. Eosin pale areas and dying neurons (between the arrowheads, see also F) in the cortex were evident and were present in both hemispheres. C) By fourteen weeks of age, all mice showed marked pathology in the cortex with vacuolization of the tissue. D) At sixteen weeks of age, the affected regions were larger and included not only the cortex, but also the inferior hippocampus (see also G). E) By eighteen weeks of age, the surviving mice had extreme tissue loss and vacuolization. F) At early stages (twelve weeks) dying neurons identified by their eosin-positive cell bodies and condensed nuclei (black arrowhead) were evident abutting regions of apparently healthy neurons (white arrowhead) indicating a recent insult and consistent with the regional damage of an ischemic event. The image is a higher magnification view of the regions boxed in B, rotated 90 degrees clockwise. G) The hippocampus of sixteen week *dcr* animals also shows dying neurons in the CA regions of the hippocampus (CA3 shown) although the dentate gyrus (at right) remains relatively unaffected. H) The dorsal medial cortex is unaffected at sixteen weeks of age in all five mice examined. I) By eighteen weeks of age, the dorsal cortex is also involved, and eosin-pale, vacuolated areas are visible. Note H and I are more rostral than other sections, the affected dorsal medial cortical regions were consistently seen rostrally to the inferior lateral cortical regions (see also figure 2).

In evaluating the onset and time course of the decrepit phenotype, it is clear that the disease is progressive (Figure 3A–E). In a systematic evaluation, zero of five mice in their tenth week of age showed pathology in the ventral cortex. Four out of five mice in the twelfth week of age showed an eosin-pale, sharply bounded region of apparent ischemia in the cortex. Dying neurons, denoted by their eosin-positive soma and condensed nuclei, were still present in this region suggesting a recent tissue insult (Figure 3F). By the fourteenth week of age, all mice were identifiable by histology (5 of 5), and the afflicted regions were larger and mostly devoid of neurons. By the 16th week, additional brain areas such as the inferior hippocampus were also affected. By the eighteenth week of age, four of four mice examined had large regions of completely vacuolated tissue in the inferior cortex and hippocampus. The dorsal cortex more rostrally was also affected by this age, although it was not affected in any of the five mice examined at sixteen weeks of age (Figure 3H, I). The affected regions of the brain were always roughly bilateral, and the pattern and severity were not markedly different in the different hemispheres.

### Cerebrovascular anatomy

**Figure 4:**
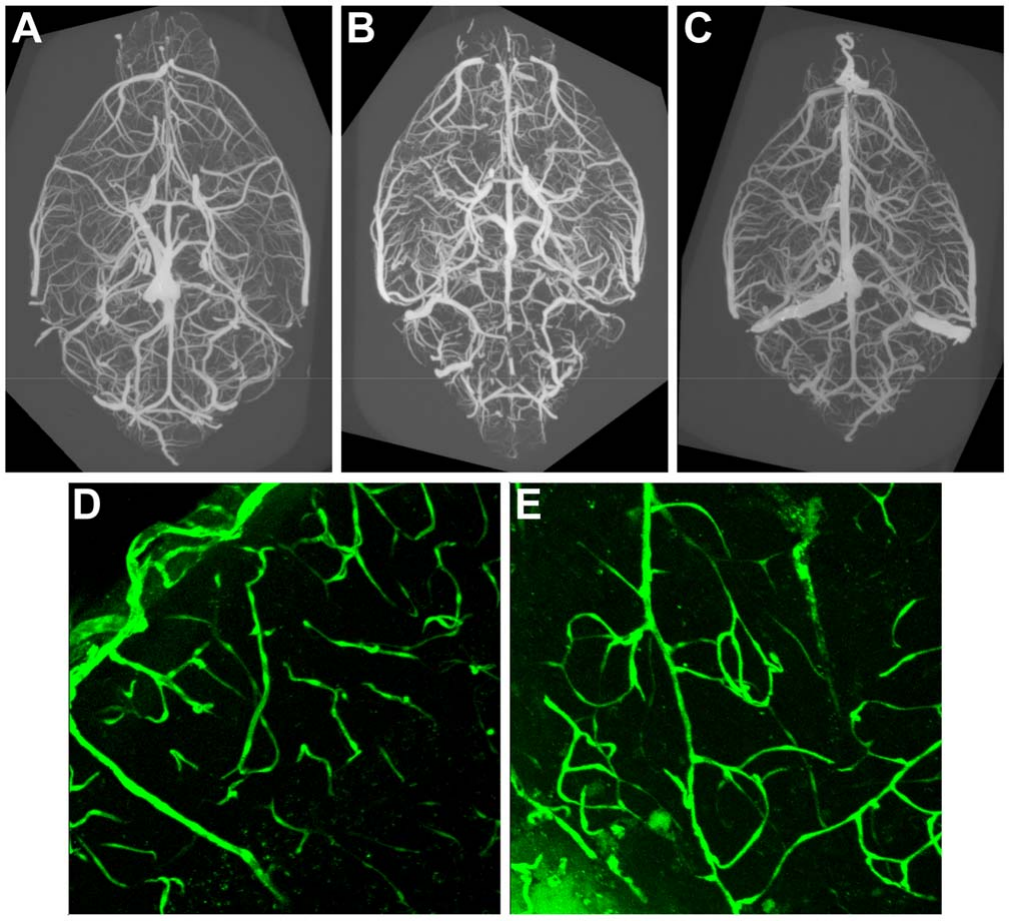
Vascular anatomy in *dcr* mice. A–C) The vasculature of control (A) and two affected *dcr* (B, C) mice at 100 days of age was reconstructed using micro-CT imaging. A and B are projections viewed ventrally, C viewed dorsally. The reconstructions presented are projections, but data can be viewed in three dimensions and rotated to examine anatomy of the vasculature. No marked changes in vascular anatomy were seen in the mutant animals. D, E) Smaller penetrating vessels and capillaries of the brain were examined in coronal sections using a GFP transgene to label endothelial cells. D) A projection of a 50 µm vertical confocal reconstruction, the pial surface is in the upper left. E) Confocal analysis of *dcr* mice did not reveal a marked change in the small vessels of the brain. The pial surface in E is just out of the field at the top left, the autofluorescence in the lower left is the necrotic tissue in the cortex.

The anatomy of the cerebral vasculature in the decrepit mice was examined using a variety of methods. First, we used micro-CT reconstruction to examine the brain vasculature at a resolution of 20 microns. Mice were anesthetized and perfused systemically through the left ventricle with 4% buffered formalin followed by Microfil, a radio-opaque latex resin. The brain was then dissected free from of the skull and embedded in agarose for imaging. Reconstruction of the vessels did not reveal any major anatomical abnormalities (Figure 4A–C). The middle and anterior cerebral arteries supply the regions of the brain that are affected, and the areas are close to watershed zones of perfusion. However, the pathology (complete vacuolization of the tissue) is most consistent with ischemia and not a hypoperfusion and resulting hypoxia, which should instead result in a glial scar. Furthermore, other organs sensitive to low blood flow, such as the renal papillus, were unaffected (not shown, n = 5). Interestingly, after the mice were moderately affected (approximately 100 days of age or greater), they became difficult to perfuse with fixative or resin. This change was not restricted to the brain, organs such as the kidneys and liver also filled poorly. If perfusion with resins was performed without prior fixation, the vessels appeared fragile and the resins permeated the tissue rather than simply filling the vasculature; however, tracers such as Evan's Blue did not leak from the vasculature into the brain parenchyma (not shown). Based on these qualitative differences in the ability to perfuse the vasculature, we suspect that vessels throughout the body are affected by the *dcr* mutation, although dysfunction resulting in ischemia and tissue death is restricted to the nervous system.

Smaller penetrating blood vessels and capillaries in the cortical regions bordering the infarcts were visualized by breeding a Tie2-GFP transgene that expresses GFP specifically in endothelial cells into the decrepit background. Coronal brain slices were then imaged using a confocal microscope to produce 50 µm reconstructions through the inferior cortex (Figure 4, D, E). No marked changes in vessels were observed.

### Decrepit genetics

**Figure 5:**
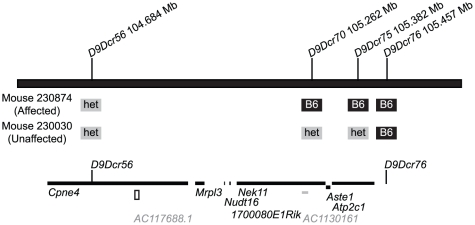
The *dcr* interval. The *dcr* locus maps between marker *D9Dcr56* and *D9Dcr76*. The spacing megabase positions of these markers and two other markers within the interval are shown. Examples of genotypes defining the proximal (*D9Dcr56*) and distal (*D9Dcr76*) ends of the region are shown. Mice homozygous for C57BL/6 markers in the region are affected (*dcr/dcr* homozygotes) whereas mice heterozygous or homozygous for BALB/c markers through the region are unaffected. Both ends of the interval are confirmed by at least three independent recombination events. The position of the protein coding genes within the interval is shown below. Gene symbols are at the proximal end of the features. The noncoding miRNA (*AC117688*.1) and the processed transcript (*AC1130161*) are also shown.

The *dcr* mutation arose spontaneously in a C57BL/6J (B6)-background strain carrying 2 independent congenic segments and was then transferred to a homogeneous B6 background. Male and female mice were equally affected and the phenotype did not segregate with either congenic segment. To map *dcr*, an affected (presumed homozygous) B6 male was bred to BALB/cByJ females and their F1 progeny were intercrossed to produce an F2 generation that yielded affected offspring at the anticipated frequency (12/53). This cross indicated that *dcr* acts as a recessive allele segregating as a single locus and that the decrepit phenotype is not substantially affected by genetic background variation. A low-density genome scan using 103 informative SSLP *Mit* markers spanning the mouse autosomes was performed on ten affected mice and twelve unaffected F2 littermates. Chromosome 9 showed linkage with the mutation, with all ten affected mice being homozygous for the B6 allele of *D9Mit182* (101.431 Mb), while no unaffected mice were homozygous for B6 at that marker (Chi square test 1.4e^−6^). The position was confirmed and limited to an interval between *D9Mit53* and *D9Mit116* (104.6 to 108.5 Mb) with additional flanking markers, the analysis of additional F2 mice, and a mapping backcross using an NOD/LtJ mapping partner. Higher resolution mapping was performed using a congenic reduction approach in which *dcr* was opposed a BALB/c segment from chromosome 9 congenic B6.C-*H7^b^*/(47N)SnDcr mice. The analysis of >2000 meioses narrowed the interval to 772, 552 bp on chromosome 9, between 104684730 and 105457282 bp (*D9Mit53*/*D9Dcr56* and *D9Dcr76*) (Figure 5). The marker *D9Dcr56* was excluded proximally from the candidate interval by 3 recombinant mice, 1 from F2 crosses and 2 from decreasing the BALB congenic interval. Distally, *D9Dcr76* was excluded from the candidate interval by 3 separate recombinant mice, all from decreasing the BALB congenic interval. Therefore, this interval appears to be suppressed for recombination and no recombinations were recorded in >1000 of the most recent meioses tested using markers 0.7 Mb apart (*D9Dcr56* and *D9Dcr75*), Additional mapping of F2 mice was performed using other strains including 129 (14 meioses), C3H (314 meioses), and SJL (72 meioses), confirming the interval on Chromosome 9, but failed to narrow it further. This indicates that the phenotype is robust on a number of genetic backgrounds, but also suggests that the reduced recombination rate is not necessarily specific to B6.BALB/c mice.

This genetic interval contains seven genes, a processed transcript, and a novel miRNA (Figure 5 and Table 1). None of the genes are obvious candidates for a vascular disease; but conversely, none can be ruled out. The region of the mouse genome shares synteny with human chromosome 3q22.1, which contains the same genes in the same relative orientations. In addition, several non-coding RNAs including a U6 splicosomal RNA are annotated in the human genome.

**Table 1:** Genes in the *dcr* genetic interval.

Gene symbol	strand	Location	Gene name
*Cpne4*	For	9:104449616- 104936874	Copine 4
*AC117688.1*	For	9:104897246- 104827349	Novel miRNA
*Mrpl3*	For	9:104955569- 104979796	Mitochondrial ribosomal protein L3
*Nudt16*	Rev	9:105031668- 105034135	Nudix (Nucleotide diphosphate linked moiety X-type motif) 16
*1700080E11Rik*	Rev	9:105045674- 105047412	Unannotated, same protein family as Nudt16
*Nek11*	Rev	9:105064797- 105297854	NIMA (Never in mitosis gene A) related expressed kinase 11
*AC1130161*	For	9:105235843- 105250710	Novel processes transcript
*Aste1*	For	9:105297722- 105308088	Asteroid homolog 1 (transcription factor)
*Atp2c1*	Rev	9:105313693- 105433477	ATPase, Ca^2+^ sequestering

The gene symbol, coding strand, base positions, and full gene names are given.

The protein coding candidate genes were examined by QPCR and northern blotting to look for differences in expression level or splicing; however, no changes were detected. The genes have also been sequenced from cDNA generated by reverse transcription of brain mRNA isolated from affected *dcr* mice. All genes amplified and no changes in sequence from the C57BL/6 reference sequence were detected. The entire 772 kb genomic interval was resequenced by array capture and high-throughput sequencing (see methods) with an average sequencing depth of 151 fold. Four SNPs were detected in comparison to the reference genome. All were confirmed but also found to be present in unaffected C57BL/6J mice. Thus, these SNPs are genuine variations from the reference sequence, but are not pathological. While coverage was not complete because repeat sequences were masked out of the array capture, only 425 bases included on the capture array were not sequenced, all of these were adjacent to masked repeat sequence and none were in exons. This sequence has been uploaded to the NCBI Sequence Reads Archive (http://www.ncbi.nlm.nih.gov/Traces/sra/sra.cgi?) and is available for further analysis (Accession # in process).

## Discussion

The *dcr* mutation appears to represent a heritable model of vascular disease, with particularly severe pathology in the brain. However, both the disease mechanism and the underlying genetic defect remain mysterious. The bilaterally-symmetric, gradually-progressive nature of the disease argues against a mechanism such as emboli or ruptured aneurisms, and no histological evidence for these mechanisms was found. Anatomical abnormalities could lead to such a reproducible and localized disease, but no obvious abnormalities were seen using either micro-CT reconstruction of larger vessels or confocal analysis of smaller penetrating vessels or capillaries. A functional defect such as vasospasm could also lead to the observed pattern of degeneration. We have not explored this mechanism extensively, but the administration of vasodilators in the drinking water between 60 and 90 days of age did not markedly alter the course of the disease. Anatomical defects such as vascular shunts could also account for such localized pathology, and could also have been challenging to detect by our methods. The presence of dilated vessels could reflect veins subjected to arterial pressures, and such shunts could result in insufficient tissue perfusion. Thus, our results are consistent with neurodegeneration arising from cerebral ischemia, but the reasons for this ischemia and for the anatomical specificity of the degeneration will require additional study.

The genetic mechanism of the *dcr* mutation is equally puzzling. The phenotype is fully penetrant, and the heritability is completely consistent with a single recessive locus mapping to Chr. 9 at 105 Mb. However, analysis of the expression level, splicing, and coding sequence of the known genes in the interval did not reveal any provocative changes. The QPCR analysis performed encompassed a larger interval, including forty genes from *Cpne4* extending distally to 2.4 Mb to *Dock3*. An apparent expression difference in *Ccg1b* was explained by a polymorphism in the primer sequence, and this gene was subsequently excluded from the genetic interval by recombination. No other changes in expression were observed, decreasing the likelihood of a mutation in non-coding regulatory element affecting the expression of a gene at a distance. No known tRNAs are found in the 0.77 Mb interval, and targeted resequencing of the entire interval should have detected any polymorphisms or deletions in the region, even in non-coding sequences.

Other chromosomal aberrations, such as inversions or copy number variations, may have been missed by the resequencing (which was aligned to the reference genome, but not assembled *de novo*). The detection of SNPs is quite straightforward, as demonstrated by the identification of four changes in comparison to the reference genome. Similarly, a deletion should have been detected as an absence of coverage relative to the reference. The insertion of novel DNA sequences, such as a retrovirus, may have been missed because such sequences would be categorized as failing to map to the interval. Similarly, inversions would also be difficult to detect with our methods and a small aberration within the interval may also account for the reduced recombination frequency observed, although inversion breakpoints may have been noted as areas of reduced sequencing coverage because the rearranged DNA would again be categorized as not mapping to the reference genome. Changes in copy number (duplications or expansions, since no deletions were detected) could also be difficult to detect, but no obvious changes in the depth of sequencing coverage were observed. To be deleterious, changes such as inversions, insertions or copy number variations would be anticipated to change gene expression levels, and no changes in mRNA levels were detected. Additional analysis such as paired end sequencing with increased read length or QPCR with multiple amplicons distributed across each gene may reveal changes that have been missed in our analysis thus far. However, at present, the underlying genetic anomaly of the *dcr* mice also remains to be determined.

As techniques for identifying rare disease loci in small patient populations improve, it is increasingly likely that a related human vascular disease will map to human Chr. 3q22, the region of shared synteny with the mouse *dcr* region. In the human genome, querying the Online Mendelian Inheritance in Man (OMIM) database did not reveal any analogous diseases mapping to the region of 3q22.

Our purpose in reporting the *dcr* locus is to make other investigators aware that an animal model of such a disease exists, which could be used to validate patient genetic mapping and any subsequent genetic lesion identified in the human genome. If a stronger linkage to human disease is determined to exist, further study of this animal model to determine the cause and to develop effective treatments may be warranted. Despite extensive characterization, robust Mendelian inheritance, and full penetrance, both the physiological basis and the genetic basis for the cerebral pathology are yet to be determined.
